# Crosstalk between polymorphic toxin-immunity systems involved in kin discrimination

**DOI:** 10.1128/mbio.00468-25

**Published:** 2025-04-15

**Authors:** Michael L. Weltzer, Tingting Guo, Daniel Wall

**Affiliations:** 1 Department of Molecular Biology, University of Wyoming 173150, Laramie, Wyoming, USA; Northern Arizona University, Flagstaff, Arizona, USA

**Keywords:** *Myxococcus xanthus*, polymorphic toxins, type VI secretion system, outer membrane exchange, kin discrimination, noncognate immunity

## Abstract

Bacterial genomes contain a surprisingly large number of toxin systems that are neutralized by specific cognate antitoxins or immunity factors. Their high abundance is even apparent in common *Escherichia coli* K12 cloning strains, which contain at least 36 toxin-antitoxin systems, while other bacteria frequently contain more. These numbers raise two key questions: why are they so numerous, and to what extent do toxin systems interact or interfere with one another? Recently in *mBio*, Wang and co-workers addressed these questions in the social bacterium *Myxococcus xanthus*, where they investigated crosstalk between four homologous toxin-immunity loci involved in kin discrimination. Here, the type VI secretion system delivers toxins into neighboring myxobacterial cells (F. Wang, J. Luo, Z. Zhang, Y. Liu, et al., mBio e03902-24, 2025, https://doi.org/10.1128/mbio.03902-24). If the target cell is clonal and expresses a complete set of cognate immunity proteins—which are not themselves transferred—the cell is protected. However, if immunity is incomplete, the cell is poisoned.

## COMMENTARY

Bacteria employ diverse toxin systems for various functions, including phage defense, stress responses, persistence, plasmid maintenance, chromosome stability, conflict systems, or other roles (1–3). In *Myxococcus xanthus*, which engages in complex social interactions, these systems primarily function in kin discrimination. This discriminatory role is crucial because, as individual cells aggregate into large social groups to perform cooperative multicellular functions, they must ensure that neighboring cells are highly related or clonal. To investigate kin discrimination, the Yue-zhong Li group previously used transposon mutagenesis to identify relevant genes by screening for mutant colonies that no longer merged with their parent strain (4). Interestingly, six loci contained insertions in T6SS immunity genes—an unexpected finding since neighboring clonal cells should poison one another in the absence of cognate immunity. For one of these toxin-immunity pairs, the researchers further demonstrated that MXAN_0050 functions as a nuclease, while the downstream gene, MXAN_0049, encoded the cognate immunity protein that binds to and neutralizes MXAN_0050 (5). In a recent study in *mBio*, they used deletion mutants in each of four immunity genes and again showed that they were viable (6).

A possible explanation for why the immunity mutants were viable is cross-immunity between homologous toxin-immunity pairs. Indeed, in biochemical assays, they demonstrated that an immunity protein bound both its cognate toxin and a divergent toxin. Additionally, they modeled interactions between this immunity protein and four toxins, suggesting that the binding surfaces were compatible and had favorable free energy binding predictions. Among these, immunities 1 and 2 are the closest homologs of the four, and as their sequence similarities decreased, their predicted and actual binding interactions also decreased. Furthermore, co-expression of different toxin-immunity (TI) combinations in a heterologous system revealed that immunity 1 protected *Escherichia coli* from poisoning by toxin 2. Finally, in mixing experiments between *M. xanthus* immunity mutants, they showed that pairs with closer sequence similarities in immunity proteins exhibited the most harmonious coexistence during vegetative growth and development.

The genes studied by Wang et al. belong to the AHH/ENDO VII nuclease (pfam14412) and Imm11 (pfam07791; DUF1629) families. Strikingly, the DK1622 strain used contains 14 *ahh* toxin loci, 5 of which belong to the T6SS, and 20 *imm11* genes, where an immunity gene always resides downstream of a toxin gene, and in some cases, there are one or two additional *imm11* genes. Additionally, for three of the experimentally tested immunity genes, homologs with a substantially higher degree of similarity (5, 6) were not tested. Thus, the potential for crosstalk, or cross-immunity, between AHH-Imm11 pairs is higher than what was tested.

**Fig 1:**
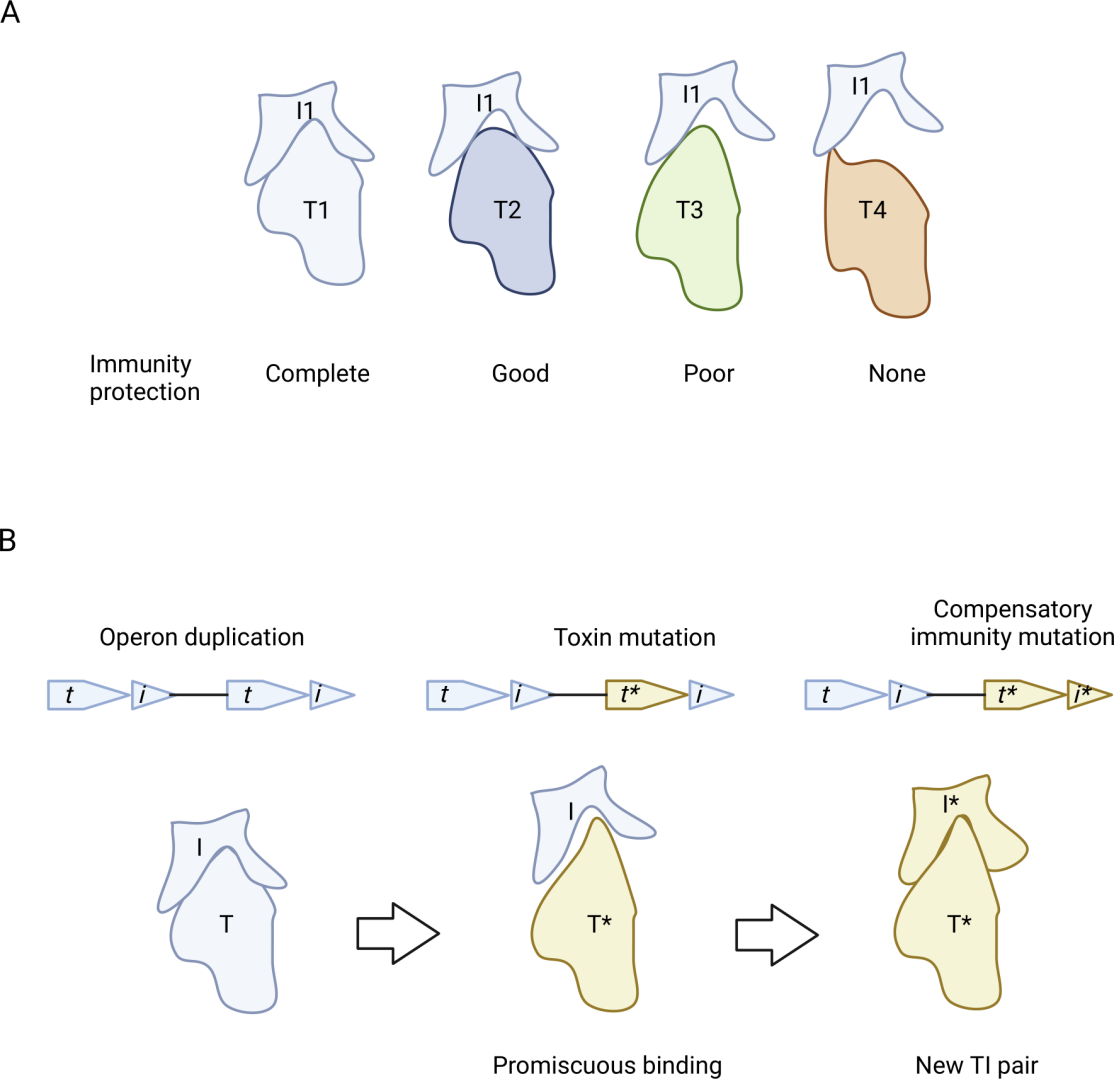
Promiscuous interactions between toxin (T) and immunity (I) proteins. (**A**) An endogenous I protein interacts with a family of toxins to different degrees. (**B**) Schematics of TI operon duplication and subsequent functional divergence through mutations. Specificity changes are often determined by a subset of interacting residues that can lead to amino acid covariance between proteins (11). Promiscuous intermediates represent an evolutionary pathway to avoid the lethal consequences of not neutralizing the toxin (12). Figure created with https://BioRender.com.

In addition to T6SS, myxobacteria have a second characterized kin discrimination system called outer membrane exchange (OME). This system uses a polymorphic cell surface receptor called TraA and the TraB cohort protein to recognize related cells by homotypic binding (7). Following recognition, cells bidirectionally exchange outer membrane content, apparently driven by transient membrane fusion, which includes a suite of polymorphic SitA lipoprotein toxins (8). Strikingly, in DK1622, the SitAI5 family contains eight *ahh-imm11* loci (9). However, between the T6SS and OME systems, the respective TI genes belong to distinct clades, and thus, crosstalk between systems is less likely. Additionally, in InterPro (10), the prototypic member of the Imm11 family is MXAN_0049 (immunity 1), which represents a clan (CL0767) that includes a family called double_CXXCG (pfam09535; TIGR02264). In turn, these immunity genes are adjacent to the SitA6 lipoprotein toxin family (pfam09533; DUF2380; TIGR02269), in which DK1622 contains nine *sitAI6* loci (9). Thus, although Imm11 and pfam09535 families share structural similarities, they share little sequence similarity, reducing the likelihood of crosstalk. However, the striking finding by Wang et al. is that cross-binding and protection against toxins occur when immunity proteins share only 46% sequence identity (6). This result builds on their prior work showing that an MXAN_0049 ortholog with 86% sequence identity provided cross-immunity (5), highlighting that relatively divergent but promiscuous interactions occur, reinforcing the need for experimental testing. Finally, although *M. xanthus* contains additional TI families, it is noteworthy that AHH-Imm11 and pfam09533-double_CXXCG family pairs play a disproportionately large role in kin discrimination, suggesting that gene/operon duplications and functional divergence were favored in myxobacteria (Fig. 1).

Crosstalk by immunity proteins provides a benefit to the host by expanding protection against the forced entry of divergent toxins involved in kin discrimination. Crosstalk also protects against mutations, which in turn drives the evolutionary diversification of TI systems (Fig. 1). In this context, it seems that selection should favor promiscuous immunity proteins while maintaining sufficient affinity for their cognate toxin (12). Although promiscuity expands host protection, the effectiveness of TI systems in kin discrimination is compromised, thus providing a counter-selection for specificity.

In summary, the study by Wang et al. highlights the role of crosstalk in polymorphic toxin systems. This has important implications for kin discrimination and raises interesting questions regarding the Imm11 family proteins in *M. xanthus*. How did Imm11 become a key player in two separate TI systems? Why do some toxin loci have multiple *imm11* genes? Do these additional *imm11* gene products crosstalk with their operonic toxin? Future studies will help unravel these questions and further shed light on how crosstalk shapes bacterial interactions and protein evolution.
